# Hepatic extracellular ATP/adenosine dynamics in zebrafish models of alcoholic and metabolic steatotic liver disease

**DOI:** 10.1038/s41598-024-58043-5

**Published:** 2024-04-03

**Authors:** Tomoko Tokumaru, Magdeline E. Carrasco Apolinario, Nobuyuki Shimizu, Ryohei Umeda, Koichi Honda, Kenshiro Shikano, Hitoshi Teranishi, Takatoshi Hikida, Toshikatsu Hanada, Keisuke Ohta, Yulong Li, Kazunari Murakami, Reiko Hanada

**Affiliations:** 1 Department of Neurophysiology, Faculty of Medicine, Oita University, Yufu, Oita Japan; 2 Division of Gastroenterology, Department of Internal Medicine, Faculty of Medicine, Oita University, Yufu, Oita, Japan; 3 Department of Cell Biology, Faculty of Medicine, Oita University, Yufu, Oita, Japan; 4 Department of Advanced Medical Science, Faculty of Medicine, Oita University, Yufu, Oita, Japan; 5 Laboratory for Advanced Brain Functions, Institute for Protein Research, Osaka University, Suita, Osaka, Japan; 6 Advanced Imaging Research Center, Kurume University, Kurume, Japan; 7 State Key Laboratory of Membrane Biology, School of Life Sciences, Peking University, Beijing, China

**Keywords:** Non-alcoholic fatty liver disease, Non-alcoholic steatohepatitis

## Abstract

Steatotic liver disease (SLD) is a burgeoning health problem predominantly associated with excessive alcohol consumption, which causes alcohol-related liver disease (ALD), and high caloric intake, which results in metabolic dysfunction-associated SLD (MASLD). The pathogenesis of ALD and MASLD, which can progress from steatohepatitis to more severe conditions such as liver fibrosis, cirrhosis, and hepatocellular carcinoma, is complicated by several factors. Recently, extracellular ATP and adenosine (Ado), as damage-associated molecular patterns, were reported to promote inflammation and liver fibrosis, contributing to SLD pathogenesis. Here, we explored the in vivo dynamics of hepatic extracellular ATP and Ado during the progression of steatohepatitis using a genetically encoded GPCR-activation-based sensor (GRAB) in zebrafish models. We established hepatocyte-specific GRAB_ATP_ and GRAB_Ado_ in zebrafish and investigated the changes in in vivo hepatic extracellular ATP and Ado levels under ALD or MASLD conditions. Disease-specific changes in hepatocyte extracellular ATP and Ado levels were observed, clearly indicating a correlation between hepatocyte extracellular ATP/Ado dynamics and disease progression. Furthermore, clodronate, a vesicular nucleotide transporter inhibitor, alleviated the MASLD phenotype by reducing the hepatic extracellular ATP and Ado content. These findings provide deep insights into extracellular ATP/Ado dynamics in disease progression, suggesting therapeutic potential for ALD and MASLD.

## Introduction

Steatotic liver disease (SLD) is a growing global public health concern. The most common etiologies for steatosis development are excessive alcohol consumption, which causes alcohol-related liver disease (ALD), and high caloric intake, which results in metabolic dysfunction-associated steatotic liver disease (MASLD), previously known as non-alcoholic fatty liver disease^1^. Both ALD and MASLD can progress to steatohepatitis and liver fibrosis, leading to cirrhosis and hepatocellular carcinoma in some cases. Risk factors for hepatocellular carcinoma have shown an epidemiological shift from virus-related to nonviral liver diseases, including ALD and MASLD^2^.

MASLD pathogenesis is complex and multifactorial^3,4^. The pathophysiological progression of MASLD involves oxidative stress, adipocytokine abnormalities, apoptosis, autophagy, and other factors related to insulin resistance caused by obesity and diabetes, as well as genetic factors^5^. Overlapping biological processes may contribute to ALD and MASLD, with liver fibrosis and prolonged inflammation having the most significant prognostic effect^1,6^. Recently, damage-associated molecular patterns (DAMPs) have been implicated in the progression of liver inflammation^7^. Among them, extracellular ATP and adenosine (Ado), released upon cellular stress or tissue injury, such as alcohol or lipid accumulation, induce sterile inflammation during the progression of ALD and MASLD^8–10^. However, the in vivo dynamics of hepatic extracellular ATP and Ado during the progression of ALD and MASLD remain unclear, warranting further investigations.

Li et al. developed genetically encoded GPCR-activation-based (GRAB) sensors that enhance the intensity of green fluorescent protein (GFP) fluorescence upon binding to specific neurotransmitters or neuromodulators^11^. These sensors can be used to analyze molecular dynamics, including those of ATP and Ado, through fluorescence measurements. Thus, GRAB sensors for ATP and Ado (GRAB_ATP_ and GRAB_Ado_) respond specifically to extracellular ATP/Ado, but not to intracellular ATP/Ado, in a concentration-dependent manner. We analyzed the extracellular ATP/Ado dynamics in the liver using zebrafish, into which this GRAB sensor was inserted.

This study was aimed at evaluating the relationship between hepatocyte extracellular ATP/Ado dynamics and the pathogenesis of ALD and MASLD in vivo using zebrafish (*Danio rerio*) models of these diseases. We generated hepatocyte-specific GRAB_ATP_ and GRAB_Ado_ zebrafish and used them to examine the phenotype of in vivo hepatic extracellular ATP and Ado kinetic changes in ALD and MASLD. We clarified the mechanistic role of hepatocyte extracellular ATP/Ado dynamics in the progression of these diseases. This study provides insights into the role of hepatic extracellular ATP and Ado in the pathogenesis of ALD and MASLD and may lead to future therapeutic strategies.

## Results

### ATP and Ado content in zebrafish liver subjected to ethanol treatment and in HepG2 culture supernatant

**Figure 1:**
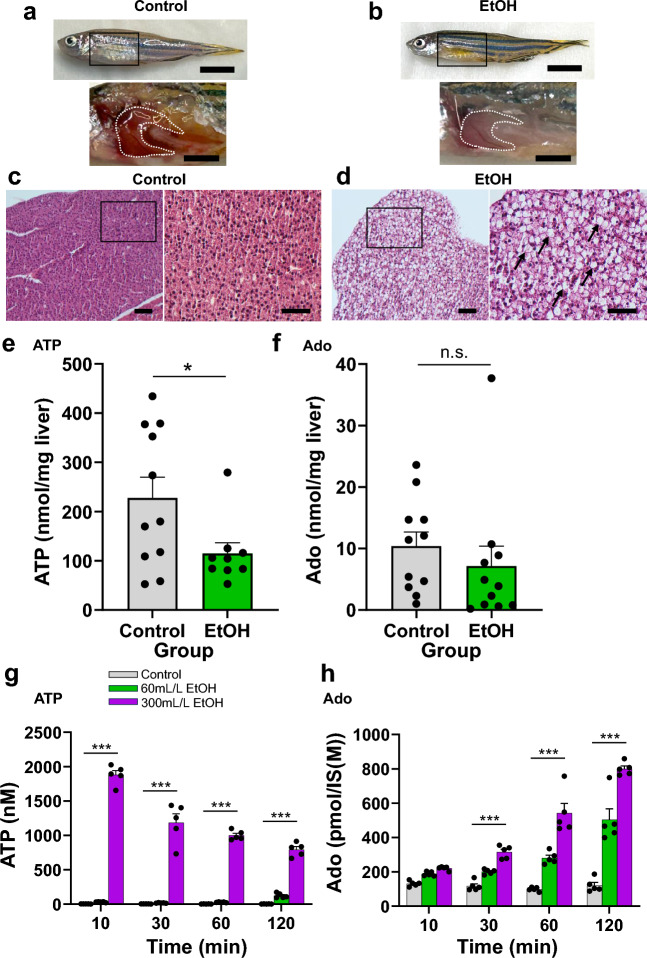
ATP and adenosine (Ado) content in the whole liver of adult zebrafish and HepG2 culture supernatant after ethanol (EtOH) exposure. (**a**,**b**) Gross appearance of zebrafish and liver (white dotted line) without (**a**) or with (**b**) 0.1% EtOH exposure for 4 weeks. Scale bars 5 mm (top panel) and 2 mm (bottom panel). (**c**,**d**) Representative hematoxylin and eosin (HE) staining of the liver sections from adult zebrafish without (**c**) or with (**d**) 0.1% EtOH exposure. Lipid droplets in the EtOH group are indicated with black arrows. Scale bar 50 µm (lower magnification) and 40 μm (higher magnification). (**e**,**f**) ATP (**e**) and Ado (**f**) content in the whole liver tissue of adult zebrafish without or with 0.1% EtOH exposure (*n* = 10, each group). (**g**,**h**) ATP (**g**) and Ado (**h**) content in HepG2 culture supernatant with or without EtOH (60 or 300 mL/L) addition (*n* = 5, each group). Data are mean ± SEM. *P* values were calculated using the Student’s *t*-test (**e**,**f**) or one-way analysis of variance (ANOVA) with Tukey’s multiple comparisons test (**g**,**h**). *P < 0.05; **P < 0.01; ***P < 0.001 vs. control. *n.s*. not significant.

Macro images of the livers of ethanol (EtOH)-treated zebrafish were whiter than those of control fish, suggesting fat accumulation (Fig. 1a,b). EtOH treatment-induced steatosis was evaluated using hematoxylin and eosin (HE) staining in vivo in untreated or ethanol-treated zebrafish (Fig. 1c,d). Successful generation of the zebrafish ALD model was confirmed by the presence of considerable adipose droplets in the livers of EtOH-treated zebrafish (Fig. 1a–d). We then analyzed whole-liver ATP and Ado content in the ALD zebrafish model. The liver ATP content in the ALD zebrafish model was significantly lower than that in control zebrafish (Fig. 1e). However, there was no difference in liver Ado content between the control and ALD zebrafish (Fig. 1f). DAMPs, including ATP and Ado, promote inflammation via adjacent Kupffer and satellite cells^12^. We examined whether EtOH treatment promoted the release of ATP from cells and altered the ATP and Ado content in the intercellular spaces using in vitro experiments. For this, we measured the content of these molecules in the supernatant of HepG2 cell culture in response to EtOH treatment, as reported previously^13,14^. There was a significant increase in ATP content 10 min after a high dose of EtOH (300 mL EtOH/L culture) was administered, followed by a time-dependent decrease (Fig. 1g); however, low and high doses of EtOH (60 and 300 mL EtOH/L culture) increased the Ado content in a dose- and time-dependent manner (Fig. 1h). In the HepG2 experiment with EtOH treatment, there were also discrepancies between intracellular and extracellular ATP and Ado kinetics (Supplementary Fig. S1). Thus, total liver ATP and Ado content, and extracellular ATP and Ado kinetics did not necessarily correlate with EtOH treatment.

### ATP and Ado content in zebrafish liver and HepG2 culture supernatant after high-fat diet

**Figure 2:**
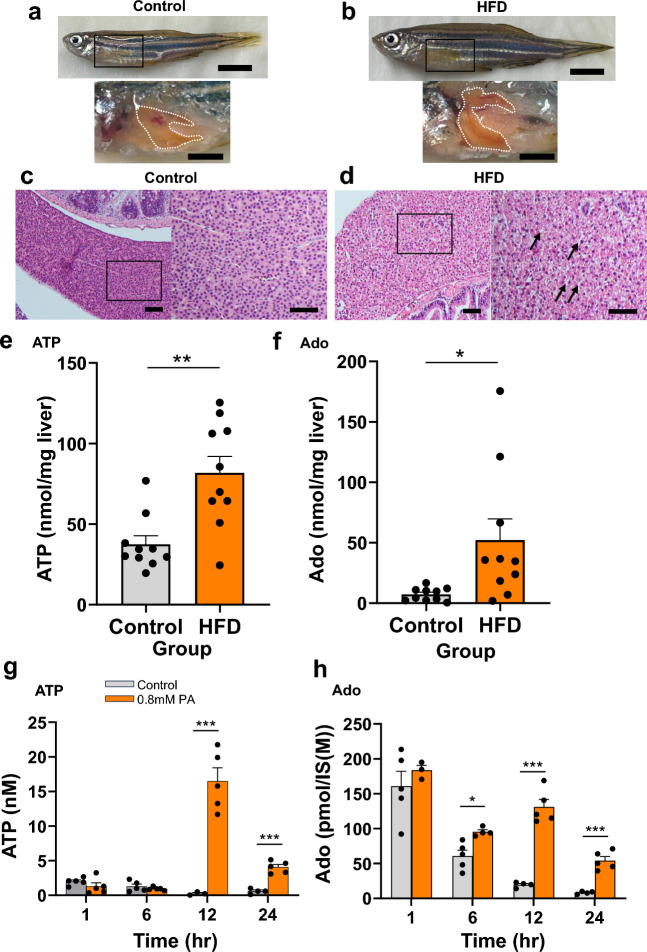
ATP and adenosine (Ado) content in the whole liver of adult zebrafish after high-fat diet (HFD) exposure and in HepG2 culture supernatant after palmitic acid (PA) treatment. (**a**,**b**) Gross appearance of zebrafish and liver (white dotted line) of zebrafish fed a control diet (**a**) or HFD (**b**) for 8 weeks. Scale bar 5 mm (top panel), 2 mm (bottom panel). (**c**,**d**) Representative hematoxylin and eosin (HE) staining of the liver sections from adult wild-type zebrafish fed a control diet (**c**) or HFD (**d**). Lipid droplets in the HFD-fed group are indicated with black arrows. Scale bar 50 µm (lower magnification) and 40 μm (higher magnification). (**e**,**f**) ATP (**e**) and Ado (**f**) content in the whole liver of adult zebrafish fed a control diet (control group) or HFD (*n* = 10, each group). (**g**,**h**) ATP (**g**) and Ado (**h**) content in HepG2 culture supernatant with or without PA (0.8 mM) treatment (*n* = 5, each group). Data are mean ± SEM. *P* values were calculated using the Student’s *t*-test (**e**,**f**) or one-way analysis of variance (ANOVA) with Tukey’s multiple comparisons test (**g**,**h**). *P < 0.05; **P < 0.01; ***P < 0.001 vs. control.

Next, we fed zebrafish a high-fat/cholesterol diet to establish the steatotic liver (MASLD-like) zebrafish model. Palmitic acid-induced fatty liver disease is a standard model of MASLD^1^. As for the ALD zebrafish model, we evaluated steatosis by examining macroimages and HE-staining of zebrafish liver (Fig. 2a–d). As expected, high-fat diet (HFD) treatment resulted in large liver sizes and many HE-stained fat droplets (Fig. 2a–d). The ATP and Ado content in the whole liver were significantly increased in the MASLD zebrafish (Fig. 2e,f). In the in vitro experiment, we measured the ATP and Ado content in the supernatant of HepG2 cells treated with palmitic acid, as reported previously^15,16^. The ATP content increased significantly (12–24 h) after palmitic acid treatment compared with that in the control (Fig. 2g). In contrast, Ado levels were higher 6 h after palmitic acid treatment and remained increased thereafter (Fig. 2h). In the HepG2 experiment with palmitic acid treatment, there were also discrepancies between intracellular and extracellular ATP and Ado kinetics (Supplementary Fig. S2).

### Establishment of GRAB_ATP_ and GRAB_Ado_ zebrafish

**Figure 3:**
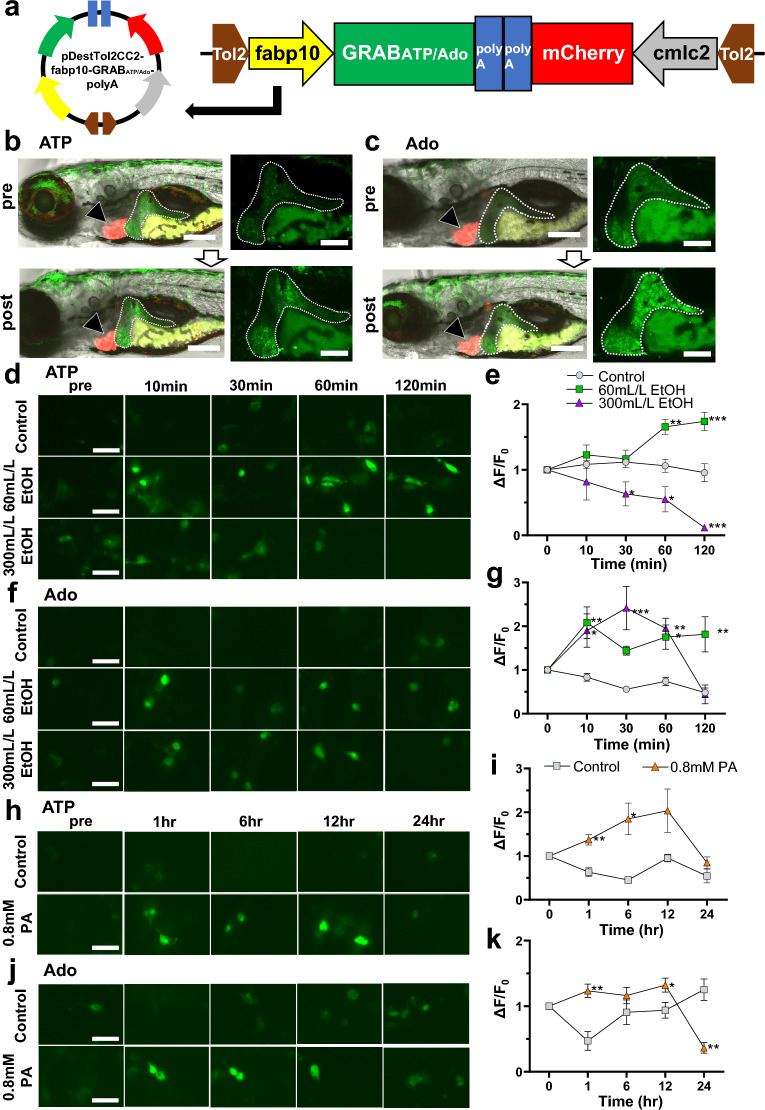
Establishment of hepatocyte-specific GRAB_ATP_ and GRAB_Ado_ zebrafish models and confirmation of GRAB sensors in HepG2 cells. (**a**) Construction of pDestTol2-CC2-fabp10-GRAB_ATP_/-GRAB_Ado_ using In-fusion cloning. (**b**) Representative images of fabp10-GRAB_ATP_ zebrafish 7 days postfertilization (dpf). Liver GFP fluorescence intensity was increased in response to 5 mM ATP treatment (bottom panel). Liver: white dotted line; mCherry-positive heart: black arrow. Scale bar 200 µm (left panel) and 100 µm (right panel). (**c**) Representative images of fabp10-GRAB_Ado_ zebrafish 7 dpf. Liver GFP fluorescence intensity was increased in response to 6 mM Ado treatment (bottom panel). Liver: white dotted line; mCherry-positive heart: black arrow. Scale bar 200 μm (left panel), 100 µm (right panel). (**d**,**e**) Representative images (**d**) and quantification of the change in GFP fluorescence intensity (**e**) in HepG2 cells transfected with pDisplay-CMV-GRAB_ATP_ with or without EtOH (60 or 300 mL/L) treatment (*n* = 5–8, each group). Scale bar 50 μm. (**f**,**g**) Representative images (**f**) and quantification of the change in GFP fluorescence intensity (**g**) in HepG2 cells transfected with pDisplay-CMV-GRAB_Ado_ with or without EtOH (60 or 300 mL/L) treatment (*n* = 5–8, each group). Scale bar 50 μm. (**h**,**i**) Representative images (**h**) and quantification of the change in GFP fluorescence intensity (**i**) in HepG2 cells transfected with pDisplay-CMV-GRAB_ATP_ with or without palmitic acid (PA; 0.8 mM) treatment (*n* = 5–8, each group). Scale bar 50 μm. (**j**,**k**) Representative images (**j**) and quantification of the change in GFP fluorescence intensity (**k**) in HepG2 cells transfected with pDisplay-CMV-GRAB_Ado_, with or without PA (0.8 mM) treatment (*n* = 5–8, each group). Scale bar 50 μm. Data are mean ± SEM. *P < 0.05; **P < 0.01; ***P < 0.001 vs. Control.

Data from the in vivo ALD and MASLD zebrafish models and in vitro studies described in previous sections indicated that the ATP and Ado content of the liver does not correlate with the extracellular ATP and Ado content, which prompted us to investigate the dynamics of extracellular ATP and Ado in ALD or MASLD to better understand their pathogenesis. We employed genetically encoded GRAB_ATP_ and GRAB_Ado_ sensors that were reported to reliably measure changes in extracellular ATP and Ado levels in the brains of living mice^17,18^. To analyze the extracellular ATP and Ado dynamics in the liver in vivo, we established hepatocyte-specific GRAB_ATP_ and GRAB_Ado_ zebrafish and used them to detect the extracellular ATP and Ado content by measuring the intensity of GFP fluorescence. These transgenic zebrafish expressed GRAB_ATP_ and GRAB_Ado_ sensor proteins using the Tol2 transposon system (Fig. 3a). First, to confirm whether GRAB_ATP_ and GRAB_Ado_ zebrafish showed a correlation in ATP and Ado content with GFP intensity in livers, we treated zebrafish with ATP or Ado as a positive control. ATP or Ado treatment clearly augmented the GFP fluorescence intensity in the liver of GRAB_ATP_ and GRAB_Ado_ zebrafish (Fig. 3b,c).

Next, we examined the effect of the GRAB_ATP_ and GRAB_Ado_ sensors in cultured HepG2 cells. Consistent with the results obtained for the ATP and Ado content in the culture supernatants (Figs. 1g,h, 2g,h), these sensors responded strongly to the application of EtOH and palmitic acid, and the GFP fluorescence intensity, which indicated extracellular ATP and Ado kinetics, was similar to that in the culture supernatants of EtOH- and palmitic acid-treated HepG2 cells (Fig. 3d–k). Furthermore, in vitro experiments also confirmed the correlation between extracellular ATP or Ado levels and fluorescence levels in HepG2 cells following ATP or Ado treatment (Supplementary Fig. S3).

### Ethanol treatment augmented GFP fluorescence intensity in the livers of GRAB_ATP_ and GRAB_Ado_ zebrafish

**Figure 4:**
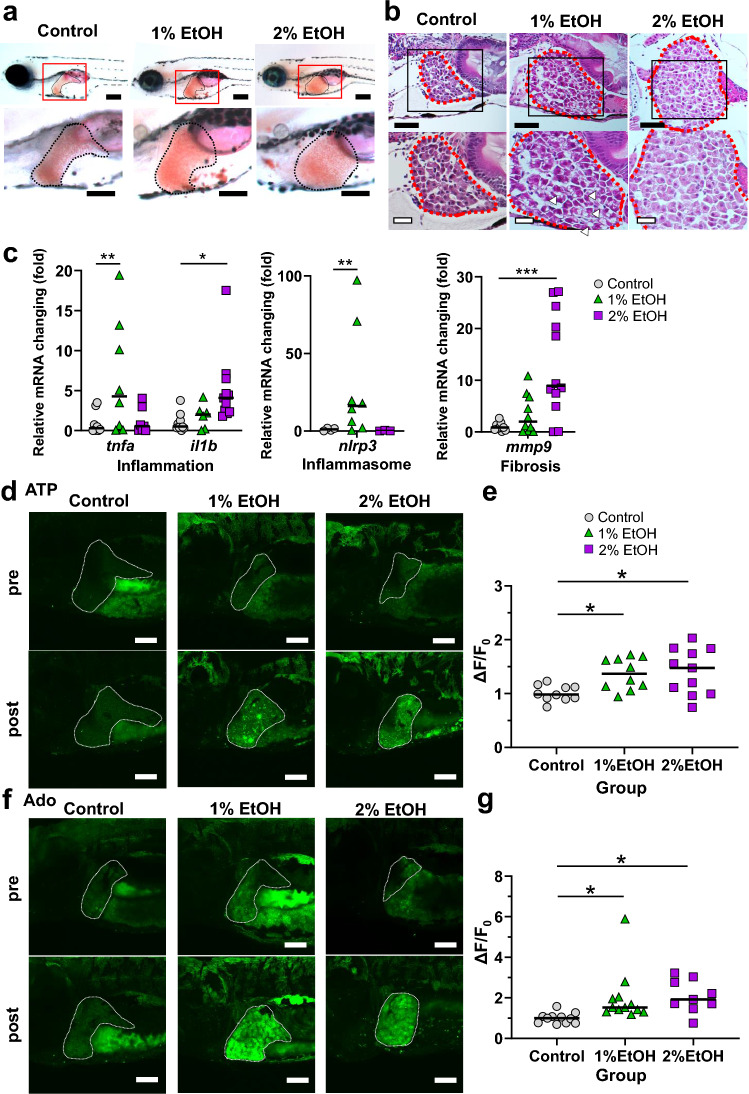
Ethanol treatment of zebrafish larvae increased the extrahepatic ATP and Ado levels. (**a**) Oil Red O staining of the liver (black line) of 6 days postfertilization (dpf) wild-type larvae with or without 1% or 2% ethanol (EtOH) exposure. Lower-magnification sagittal images (top panel) and higher-magnification images (bottom panel) are shown. Scale bar 200 µm. (**b**) Representative hematoxylin and eosin (HE)-stained images of the liver sections (red dotted line) from zebrafish larvae. Lipid droplets are seen in 1% EtOH-exposed larvae (white arrowheads). Scale bars 40 µm (top panel) and 20 µm (bottom panel). (**c**) Hepatic mRNA levels of inflammatory genes *tnfa* and *il1b*, inflammasome-related gene *nlrp3*, and fibrosis marker *mmp9* were determined in zebrafish larvae with or without 1% or 2% EtOH exposure using qRT-PCR. (**d**,**e**) Representative images of the liver of GRAB_ATP_ zebrafish larvae at 6 dpf. The GFP fluorescence intensity in the liver was increased in response to EtOH treatment (bottom panel). Liver: white dotted line. Scale bar 100 μm (**d**). Quantification of the change in the GFP fluorescence intensity in GRAB_ATP_ zebrafish larvae with or without EtOH treatment (*n* = 10, 10, 11, each group) (**e**). (**f**,**g**) Representative images of the liver of GRAB_Ado_ zebrafish larvae at 6 dpf. The GFP fluorescence intensity in the liver was increased in response to EtOH treatment (bottom panel). Liver: white dotted line. Scale bar 100 μm (**f**). Quantification of the change in the GFP fluorescence intensity in GRAB_Ado_ zebrafish larvae with or without EtOH treatment (*n* = 11, 12, 9, each group) (**g**). Data are mean ± SEM. *P < 0.05; **P < 0.01; ***P < 0.001 vs. control.

GRAB_ATP_ and GRAB_Ado_ zebrafish larvae were used to examine extracellular ATP/Ado dynamics in ALD pathology. The livers of EtOH-treated larvae showed a drastic increase in the size of Oil red O-positive regions compared with those in non-EtOH-treated larvae (Fig. 4a). HE staining also confirmed not only the increase in the size of livers but also fat droplets in larvae treated with 1% EtOH (Fig. 4b). These findings clearly showed the steatosis status and confirmed that the ALD model was established in larvae. Besides the morphological analysis, we examined the mRNA levels of proinflammatory cytokines (*tnfa* and *il1b*), the inflammasome marker (*nlrp3*), and a fibrosis-related gene (*mmp9*). The 1% EtOH treatment significantly upregulated *tnfa* mRNA levels, and the 2% EtOH treatment increased *il1b* mRNA expression compared with those in the control (Fig. 4c). The *nlrp3* mRNA levels were increased in the 1% EtOH treatment but not in the 2% EtOH treatment (Fig. 4c). The *mmp9* mRNA levels with EtOH treatment were upregulated in a dose-dependent manner compared with those in the control (Fig. 4c). Next, we examined ATP and Ado dynamics in the ALD condition using the GRAB sensor zebrafish. As indicated in representative images and quantification data, GFP fluorescence intensity in the livers of GRAB_ATP_ zebrafish treated with 1% or 2% EtOH was augmented compared with that in the no or pre-EtOH treatment group (Fig. 4d,e). Similar to the ATP dynamics, the change in GFP fluorescence intensity in the livers of GRAB_Ado_ zebrafish treated with 1% or 2% EtOH was significantly increased compared with those in the no or pre-EtOH treatment group (Fig. 4f,g). Thus, EtOH treatment increased extrahepatocyte ATP and Ado content, and we visualized these dynamics in vivo in zebrafish for the first time.

### Cholesterol treatment augmented the intensity of GFP fluorescence in the livers of GRAB_ATP_ and GRAB_Ado_ zebrafish

**Figure 5:**
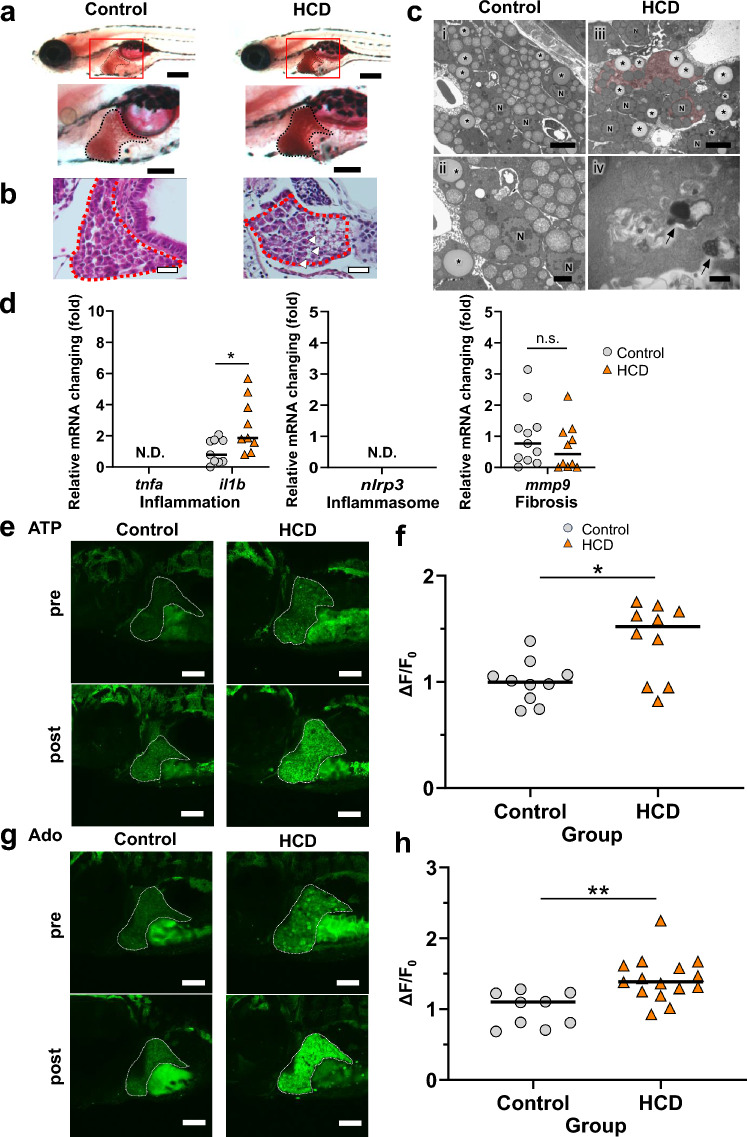
Cholesterol treatment of zebrafish larvae increased the extrahepatic ATP and Ado levels. (**a**) Oil Red O staining of the liver (black line) of 8 days postfertilization (dpf) wild-type larvae fed a normal (control) or 5% cholesterol (HCD) diet. Lower magnification sagittal images (top panel) and higher magnification images (bottom panel) are shown. Scale bar 200 µm. (**b**) Representative hematoxylin and eosin (HE)-stained images of the liver sections (red dotted line) from zebrafish larvae. Lipid droplets are seen in larvae fed a HCD (white arrowheads). Scale bar 20 µm. (**c**) Representative electron micrographs of the control and HCD groups. Lipid droplets (asterisks), glycogen accumulation (red painted area) and lysosomal phagocytosis (black arrows) are visible in the HCD group. The vacuoles often found in control samples were mitochondria. Scale bar 5.0 μm for (**i**,**iii**); 2.0 μm for (**ii**); 500 nm for (**iv**). (**d**) Hepatic mRNA levels of inflammatory genes *tnfa* and *il1b*, inflammasome-related gene *nlrp3*, and fibrosis marker *mmp9* were determined in the control and HCD groups of zebrafish larvae using qRT-PCR. (**e**,**f**) Representative images of the liver of GRAB_ATP_ zebrafish at 8 dpf. The GFP fluorescence intensity in the liver was increased in response to HCD treatment (bottom panel). Liver: white dotted line. Scale bar 100 μm (**e**). Quantification of the change in the GFP fluorescence intensity in GRAB_ATP_ zebrafish larvae with or without cholesterol treatment (*n* = 10, each group) (**f**). (**g**,**h**) Representative images of the liver in GRAB_Ado_ zebrafish larvae at 8 dpf. The GFP fluorescence intensity in the liver was increased in response to HCD treatment (bottom panel). Liver: white dotted line. Scale bar 100 μm (**g**). Quantification of the change in the GFP fluorescence intensity in GRAB_Ado_ zebrafish larvae with or without HCD treatment (*n* = 9, 15, each group) (**h**). Data are mean ± SEM. *P < 0.05; **P < 0.01 vs. control. *N.D*. not detected, *N* nucleus, *n.s*. non-significant.

We next assessed the MASLD pathophysiology in relation to extracellular ATP/Ado dynamics in GRAB_ATP_ and GRAB_Ado_ zebrafish larvae administered a 5% cholesterol diet (HCD). Oil red O staining showed that the livers of cholesterol-treated larvae increased in size and were strongly stained (Fig. 5a). HE staining showed that the number of fat droplets was increased in the HCD treatment group (Fig. 5b). To examine the steatotic livers of zebrafish larvae in more detail, we performed transmission electron microscopy (TEM) analysis. The TEM images showed increased fat droplets, glycogen accumulation, which is visible in pathological conditions, including MASLD^19,20^, and lysosomal phagocytosis, which is also observed in a lipid overaccumulation status^21^, in the livers of HCD-treated larvae but not in control larvae (Fig. 5c). These findings indicated that the steatotic liver (MASLD-like) model was established in zebrafish larvae. Next, we examined the mRNA levels of proinflammatory cytokines, inflammasome markers, and fibrosis-related genes. HCD treatment significantly upregulated the expression of *il1b*, but not other mRNAs compared with the respective levels in the control zebrafish (Fig. 5d). Excess nutrients are converted into glycogen and triglycerides, which are stored in various organs, including the liver, leading to the progression to fatty liver^22^. We examined the ATP/Ado dynamics under MASLD-like conditions using the GRAB sensor zebrafish. As shown in the representative images and quantification data, the GFP fluorescence intensity in the livers of GRAB_ATP_ zebrafish was augmented after HCD treatment compared with that in the no or pre-HCD treatment group (Fig. 5e,f). Similar to the ATP dynamics, the GFP fluorescence intensity in the livers of GRAB_Ado_ zebrafish was significantly increased after HCD treatment compared with that in the no or pre-cholesterol treatment group (Fig. 5g,h). Thus, HCD treatment also increased extracellular ATP and Ado content, and we could visualize these dynamics in vivo in zebrafish.

### Clodronate prevents MASLD-like pathogenesis progression by decreasing hepatic extracellular ATP and Ado levels

**Figure 6:**
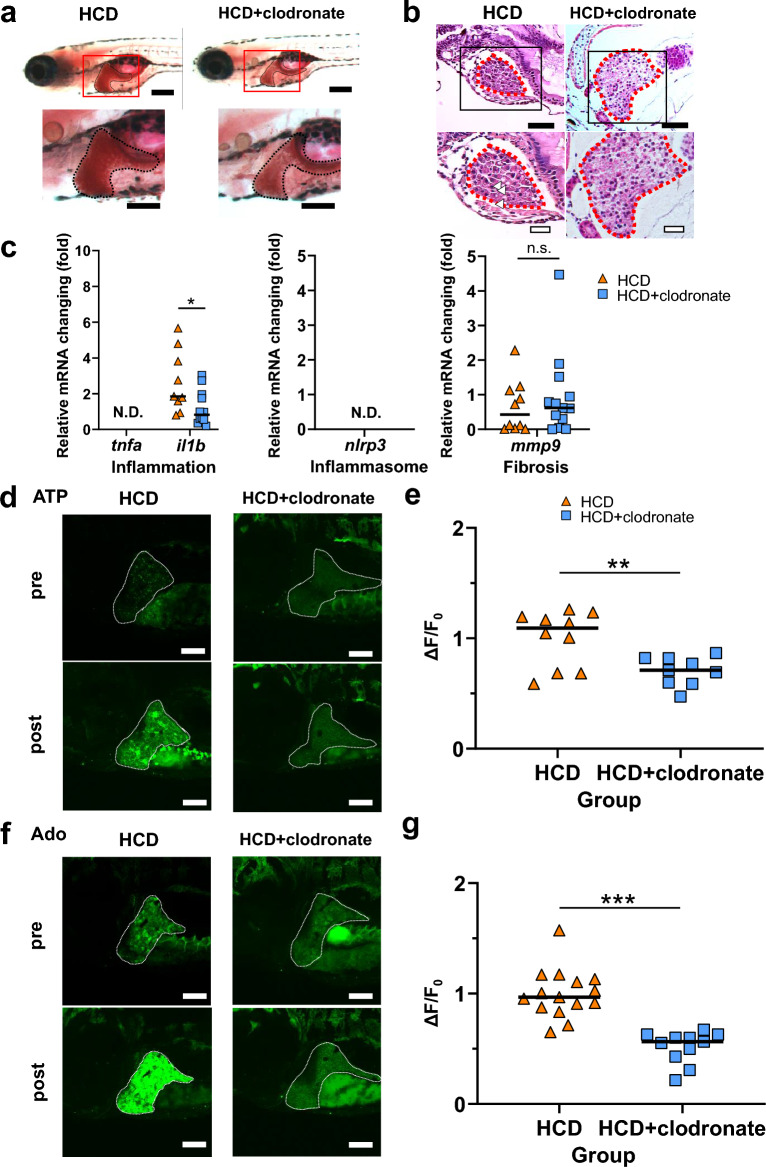
Clodronate improved the MASLD condition correlating with the reduction in extrahepatic ATP and adenosine (Ado) levels. (**a**) Oil red O staining of the liver (black line) of 8 days postfertilization (dpf) larvae fed a 5% cholesterol diet (HCD) with or without clodronate treatment. Lower-magnification sagittal images (top panel) and higher magnification images (bottom panel). Scale bar 200 µm. (**b**) Representative hematoxylin and eosin (HE)-stained images of the liver sections (red dotted line) of zebrafish larvae are shown. Lipid droplets are seen in HCD-fed larvae (white arrowheads). Scale bars 40 µm (top panel) and 20 µm (bottom panel). (**c**) Hepatic mRNA levels of inflammatory genes *tnfa* and *il1b*, inflammasome-related gene *nlrp3*, and fibrosis marker *mmp9* were determined in HCD groups of zebrafish larvae with or without clodronate treatment using qRT-PCR. (**d**,**e**) Representative images of the liver in GRAB_ATP_ zebrafish larvae at 8 dpf in the HCD group with or without clodronate treatment. High GFP fluorescence intensity in the liver induced by HCD was reduced by clodronate treatment (bottom panel). Liver: white dotted line. Scale bar 100 μm (**d**). Quantification of the change in the GFP fluorescence intensity in GRAB_ATP_ zebrafish larvae without or with HCD treatment (*n* = 10, 9, each group) (**e**). (**f**,**g**) Representative images of the liver in GRAB_Ado_ zebrafish larvae at 8 dpf in the HCD group with or without clodronate treatment. High GFP fluorescence intensity in the liver induced by HCD was reduced by clodronate treatment (bottom panel). Liver: white dotted line. Scale bar 100 μm (**d**). Quantification of the change in the GFP fluorescence intensity in GRAB_Ado_ zebrafish larvae without or with HCD treatment (*n* = 15, 11, each group) (**e**). Data are mean ± SEM. *P < 0.05; **P < 0.01; ***P < 0.001 vs. control. *N.D*. not detected, *HCD* high-cholesterol diet, *n.s*. non-significant.

Clodronate, an inhibitor of vesicular nucleotide transporter (VNUT) that is essential for vesicular ATP storage and subsequent ATP release to the extracellular space^23–25^, ameliorates steatosis in mice^26^. However, there is no direct evidence for the involvement of clodronate in hepatic ATP/Ado dynamics in an in vivo MASLD-like model. Therefore, to investigate whether clodronate improves MASLD-like pathogenesis in vivo, which correlates with the extracellular hepatic ATP/Ado dynamics, we administered clodronate to GRAB_ATP_ and GRAB_Ado_ zebrafish larvae with MASLD-like conditions induced using HCD treatment. Clodronate improved steatosis, as evident by reduced Oil Red O-stained regions in the liver compared with those in the untreated MASLD-like model (Fig. 6a). HE staining also revealed that the number of lipid droplets decreased with clodronate treatment (Fig. 6b). At the molecular level, *il1b* mRNA levels in the liver were reduced after clodronate treatment compared with those in the untreated MASLD-like model (Fig. 6c). We evaluated ATP and Ado dynamics in the liver of GRAB sensor zebrafish larvae under these conditions. The GFP fluorescence intensity in the livers of GRAB_ATP_ zebrafish was decreased after clodronate treatment compared with that in the untreated MASLD-like model (Fig. 6d,e). Similarly, the GFP fluorescence intensity in the livers of GRAB_Ado_ zebrafish was significantly decreased after clodronate treatment compared with that in the untreated MASLD-like model (Fig. 6f,g). These data indicate that clodronate improved the MASLD-like model by decreasing the hepatocyte extracellular ATP and Ado levels.

## Discussion

In this study, we established a unique zebrafish model that can be used to visualize and detect the hepatic extracellular ATP/Ado dynamics using a GRAB sensor. Using this zebrafish model, we obtained direct evidence for a correlation between the pathological progression of ALD and MASLD and the hepatocyte extracellular ATP/Ado dynamics in zebrafish. EtOH or overnutrition treatments resulted in steatosis, as revealed by the morphological analysis of adult zebrafish. However, no clear and consistent correlation between the fatty liver and whole-liver ATP or Ado content was noted. In addition, changes in the dynamics of whole-liver ATP or Ado content in the in vivo steatosis model and in the extracellular ATP or Ado content in in vitro HepG2 cells did not match. Therefore, we established liver-specific GRAB_ATP_ or GRAB_Ado_ zebrafish and demonstrated that EtOH and cholesterol treatments significantly increased the GFP florescence intensity in livers in response to extracellular ATP or Ado content in these models. Clodronate, which was reported to ameliorate steatosis^26^, improved the MASLD-like status by reducing the hepatocyte extracellular ATP and Ado content. We were able to visualize correlations in the hepatocyte extracellular ATP/Ado dynamics and pathological progression of ALD and MASLD in zebrafish in vivo.

Hepatic inflammation is implicated in various metabolic liver diseases, such as ALD and MASLD^27^. Recently, innate immunity has been suggested to counter not only pathogens but also endogenous antigens^10^. This type of inflammation is called “sterile inflammation”. Hepatic sterile inflammation is initiated by DAMPs such as ATP or Ado, and extracellular ATP and Ado levels play a pivotal role in the pathogenesis of steatotic liver disease^7^. Vesicular ATP release has been suggested to be a risk factor for various conditions and diseases associated with metabolic syndromes, including steatotic liver diseases^28–32^.

As previously reported, we found that EtOH^33,34^ or over-nutrition^35,36^ induced steatosis. In our study, the ATP and Ado content in whole livers did not completely corroborate the in vitro data for the extracellular ATP and Ado content in the culture supernatant of HepG2 cells subjected to EtOH or overnutrition treatment. Thus, the hepatocyte extracellular ATP and Ado levels showed unique kinetics, different from those in the liver as a whole. We believe that these kinetics may be involved in the pathogenesis of ALD or MASLD. The molecular mechanisms leading to steatotic hepatitis are different in ALD and MASLD. EtOH consumption induces oxidative reactions as a consequence of a dehydrogenase-mediated increase in the NADH/NAD^+^ ratio in cytoplasms and mitochondria, resulting in a hypermetabolic state^37^. This impedes mitochondrial β-oxidation of free fatty acids and drives the accumulation of intracellular lipids within the hepatocytes^38^. In this study, we found that EtOH treatment induced steatosis in both adult and larval zebrafish. Previous in vitro studies have shown that administration of EtOH or nutritional overload increased the release of ATP or Ado into the extracellular milieu^8,39^. Our in vitro study demonstrated that the Ado, but not ATP, content in the HepG2 culture supernatant was increased by EtOH treatment. Notably, EtOH treatment decreased the ATP content in the HepG2 culture supernatant in a time-dependent manner. This phenomenon may be because the released ATP is immediately degraded into adenosine diphosphate (ADP) or Ado^40^. Zebrafish larvae treated with EtOH developed steatosis, as evident upon morphological examination. At 1%, EtOH increased the levels of *tnfa* mRNA, which is a proinflammatory cytokine, and of *nlrp3* mRNA, which is the main component of the inflammasome. In addition, 2% EtOH increased the levels of *il1b* mRNA, a proinflammatory cytokine induced by inflammasome activation, and *mmp9* mRNA, a marker of fibrosis. These data indicated that 1% EtOH treatment mimics the early stage of a fatty liver condition, inducing *tnfa* and *nlrp3* mRNA expression. In addition, the severity of fatty liver appeared to be greater in the 2% EtOH treatment than in the 1% EtOH treatment. This was due to increased levels of *il1b* mRNA, which is expressed after the activation of inflammatory markers, and of *mmp9* mRNA, which is a marker of fibrosis. EtOH treatment increased the GFP fluorescence intensity in GRAB_ATP_ and GRAB_Ado_ zebrafish relative to that in the control in a dose-dependent manner under the pathological ALD-like conditions.

In the case of insulin resistance, excessive fat accumulation due to overnutrition induces hepatic deposition of triglycerides because of the increased release of free fatty acids from adipocytes, which leads to steatosis^41^. We found that excessive nutritional intake resulted in steatosis in both adult and larval zebrafish. The ATP and Ado content in the whole liver tissue of adult MASLD-like zebrafish increased compared with that in control zebrafish. In the in vitro experiment, palmitic acid treatment significantly decreased the ATP content in the supernatant of HepG2 culture, except at 12 h after the treatment. In contrast, the Ado content in the supernatant was consistently higher in the liver after palmitic acid treatment. This may be due to a reflection of the intracellular ATP content, or ATP degradation may have progressed prior to measurement. Since the expression of ATP and ADP-degrading enzymes has been reported to increase in inflammatory conditions^42^, including the pathogenesis of steatohepatitis, these data indicate that the released ATP might have been immediately degraded to ADP or Ado^40^. Cholesterol loading in zebrafish larvae produced fatty liver morphology but mildly increased the levels of only the *il1b* mRNA in our assessment of the expression of genes related to inflammation, inflammasome activation, and fibrosis. We further confirmed the MASLD-like status using electron microscopy. As expected, the images revealed glycogen granules and fat droplets, which are characteristic of fatty liver^19^, confirming the pathogenesis of MASLD. Analysis using GRAB_ATP_ or GRAB_Ado_ zebrafish under this condition revealed an increase in the GFP fluorescence intensity in the HCD treatment compared with that in the controls. These data also indicated that extracellular ATP and Ado levels are closely associated with the pathological progression of MASLD. ATP is released via VNUT-mediated vesicular release from hepatocytes upon EtOH treatment^43^ or nutritional overload^32^. VNUT is expressed in various ATP-secreting cells and is able to transport a wide variety of nucleotides in a vesicular membrane potential-dependent manner, functioning in vesicular storage and release of ATP and resulting in purinergic transmission^44^. This vesicular ATP release, along with degraded Ado, constitutes a risk for conditions and diseases associated with metabolic syndromes, including MASLD. Thus, VNUT is a key molecule in the initiation of purinergic signalling involving ATP/Ado, the DAMPs, for immunological metabolic disruption and function^30^. Hepatocytes release ATP in a VNUT-dependent manner, inducing hepatic insulin resistance and inflammation^32^. Furthermore, hepatic inflammation and fibrosis were markedly reduced in an HFD-induced NASH model in VNUT-knockout mice, whereas clodronate improved the pathological condition of MASLD^23^. Mice lacking the P2X7 receptor, which is a purine receptor particularly involved in inflammation, are resistant to alcoholic and dietary steatohepatitis^43,45^. In addition, Ado promotes liver fibrosis^8^, and caffeine^46^, which has antagonistic effects on adenosine receptors, reduces the development of liver fibrosis and liver disease-related diseases. Based on this evidence, inhibition of extracellular purinergic signaling mediated by VNUT could prevent the progression of MASLD via the reduction of extracellular ATP and Ado content. In consonance with previous reports, we found that treatment with clodronate, a VNUT inhibitor, improved fatty liver pathology, and *il1b* mRNA levels enhanced expression. As hypothesized, clodronate significantly reduced the GFP fluorescence intensity, indicative of extracellular ATP and Ado levels, in GRAB zebrafish models with MASLD-like conditions. These data also show that the hepatocyte extracellular ATP and Ado levels are accurate indicators of the pathological progression of ALD and MASLD.

We observed a clear correlation between fatty liver progression and GFP fluorescence intensity, indicative of extracellular ATP and Ado levels, using the GRAB sensors. However, no strong association with cytokine levels, inflammasome activation, or fibrosis was noted, which could be due to the fact that detection of extracellular ATP and Ado levels using the GRAB sensor is more immediate and sensitive to the pathological progression and may be an indicator of the earlier disease status.

We showed that the hepatocyte extracellular ATP/Ado dynamics reliably correlate with the pathogenesis of ALD-like and MASLD-like conditions. However, the exact sources of extracellular ATP and Ado remain unclear. It is also known that the released ATP and ADP, or Ado, produced by ATP degradation bind to many types of purine receptors that are intricately involved in biological and pathological processes^42,47^. The binding of ATP or Ado to purine receptors may play a complex role in biological and pathological processes; however, details of the molecular mechanisms mediating purinergic systems in the pathogenesis of ALD or MASLD remain unclear. Thus, the detailed molecular mechanisms mediating the ATP/Ado dynamics in target cells require further investigation.

In summary, we show that extracellular ATP/Ado dynamics correlate with the pathological progression of ALD and MASLD and play an important role in their pathogenesis using liver-specific GRAB_ATP_ and GRAB_Ado_ zebrafish models established by us. We further elucidated new pathophysiological mechanisms underlying ALD and MASLD, which may lead to novel therapeutic strategies employing extracellular ATP/Ado dynamics as an accurate indicator of steatosis progression.

## Methods

### Zebrafish maintenance

All zebrafish (AB strain; ZFIN, Eugen, OR) were raised under a 14 h-light:10 h-dark cycle at 28–29 °C. Unfertilized eggs and chorions, posthatching, were removed with care. Embryos were harvested and kept at 28.5 °C. All experimental animal procedures were performed in accordance with the institutional and national guidelines and regulations. The study was carried out in compliance with the ARRIVE guidelines. All zebrafish protocols were approved by the Institutional Review Board of Oita University (approval number 190301).

### Diets

The control diets were Hikari Labo 130 (Kyorin, Hyogo, Japan) and artemia (brine shrimp eggs; A&A Marine Goods, Tilbury, Canada). The energy content of Hikari Labo 130 was 3 kcal/g, with 10.2% of the calories from fat and 52.9% from protein, and that of artemia was 4.4 kcal/g, with 22% of the calories from fat, 44% from protein, and 16% from carbohydrates. The energy content of HFD (egg yolk powder; Yoshigai, Fukuoka, Japan) was 6.7 kcal/g, with 59% of the calories from fat, 32% from protein, and 2% from carbohydrates.

### Ethanol treatment of adult zebrafish and larvae

For experiment with the adult zebrafish, two groups of 10 wild-type male zebrafish (2–4 months postfertilization; mpf) were reared in an isolated fish tank containing 1 L of water with or without 0.1% (v/v) EtOH for 4 weeks. Fresh water, with or without EtOH, was refreshed daily. The zebrafish were fed regular food (Hikari Labo 130; Kyorin, 20 mg/fish, the tested amount of food that can be completely consumed within 3 h) once daily. They were starved for 24 h before sacrifice.

For experiments with larvae, four days postfertilization (dpf), larvae were treated with 0%, 1%, or 2% EtOH for 48 h by adding absolute EtOH to E3 medium (10 mM HEPES pH 7.2, 5 mM NaCl, 0.17 mM KCl, 0.33 mM CaCl_2_·2H_2_O, 0.33 mM MgSO_4_). They were starved during this period.

### High-fat diet treatment of zebrafish larvae and adults

For experiments with adult zebrafish, 2–4 mpf wild-type adult zebrafish were fed a standard diet (artemia; 5 mg/fish) or HFD (a combination of artemia; 5 mg/fish and egg yolk powder; 30 mg/fish) for 3 h once a day for 8 weeks following the previous protocol^36^. For experiments with larvae, zebrafish larvae in the standard diet or HCD group were fed the control (Hikari Labo 130; Kyorin, 2 mg/fish) or HCD (5% cholesterol added to the control diet; FUJIFILM Wako Pure Chemical Corporation, Osaka, Japan) diet following the schedule from 5 to 8 dpf. Both the adults and larvae were starved for 24 h before sacrifice.

### Treatment larvae zebrafish with clodronate

Zebrafish larvae fed HCD (as described above) were raised with or without exposure to 125 µg/mL clodronate (Tokyo Chemical Industry, Tokyo, Japan) following a schedule from 5 to 8 dpf. Water with/without clodronate was exchanged after every feeding.

### Liquid chromatography-mass spectrometry (LC–MS/MS) analysis of ATP and Ado content in the liver of adult zebrafish

Adult wild-type zebrafish (3–5 mpf for the after-EtOH treatment protocol or 5–7 mpf for the after-HFD treatment protocol) were anesthetized and their livers dissected, which were washed in phosphate-buffered saline (PBS), and kept in – 80 °C until LC/MS analysis. The samples for LC/MS were prepared as described previously^48^. The pellets of the supernatants were dissolved in 20 µL of deionized water, and 10 µL of each sample was injected into an LC/MS system (ACQUITY UPLC H-Class, AQUITY QDa; Waters, Milford, MA). The internal (0.125, 0.25, 0.5, and 1.0 mM, 2-isopropylmalete) and external (6.25, 12.5, 25, 50, and 100 µM NTPs for ATP measurement; 5 nM, 50 nM, 500 nM, 5 µM, and 50 µM Ado solution for Ado measurement) standards were injected together. Protein concentrations were determined using the Bradford assay with bovine serum albumin as the standard protein. The amount of free NTPs was normalized against total protein concentration.

### Cell culture experiments

Human hepatoblastoma HepG2 cells were purchased from the American Type Culture Collection (ATCC). HepG2 cells were cultured in RPMI Medium 1640 (Thermo Fisher Scientific, Waltham, MA, USA), supplemented with 10% fetal bovine serum (Sigma-Aldrich, St. Louis, MO, USA). The cells were incubated at 37 °C in a humidified atmosphere with 5% CO_2_.

For transfection, pDisplay-CMV-GRAB_ATP_ and pDisplay-CMV-GRAB_Ado_ plasmids were constructed using PCR with KOD plus Neo (Toyobo, Osaka, Japan) and In-Fusion Snap Assembly Master Mix (Takara, Otsu, Japan). The GRAB_ATP_ and GRAB_Ado_ sequences were amplified using PCR with the following primers listed in Table 1.

**Table 1:** Primers used for genotyping in this study.

Gene	Primer sequence (5′–3′)
Primers for GRAB_ATP_ sequence analysis	Forward: GGGGATGCCATGTGTAAACT Reverse: CACGCTCAGGTAGTGGTTGT
Primers for GRAB_Ado_ sequence analysis	Forward: ACAACCACTACCTGAGCGTG Reverse: AGATGGTGCGCTCCTGGATG

HepG2 cells at 70% confluency were transiently transfected with the pDisplay-CMV-GRAB_ATP_ or pDisplay-CMV-GRAB_Ado_ plasmid using Lipofectamine™ 3000 (Thermo Fisher Scientific). After 48 h of transfection, the cells were treated with EtOH, DMSO, or palmitic acid (FUJIFILM Wako Pure Chemical Corporation) for the indicated time periods to perform the assays. The cells were viewed and photographed using a Biorevo BZ-9000 fluorescence microscope (Keyence, Osaka, Japan). The fluorescence was measured with a BZ-II analyzer (Keyence).

### Quantitation of ATP in cell culture supernatant

To measure ATP levels in cell culture supernatants, HepG2 cells were treated with the medium alone, or with EtOH at 60 or 300 mL/L, or DMSO at 0.8 mM, and palmitic acid at 0.8 mM of culture for the scheduled time in a 24-well tissue culture plate. The cell culture medium was collected and immediately placed on ice. ATP concentrations were measured using an ATP-Assay-Kit (Dojindo laboratories, Kumamoto, Japan) and a luminometer, following the manufacturer’s instructions.

### Quantitation of Ado in cell culture supernatant using LC–MS/MS

Levels of Ado were measured using LC-MS/MS as described previously^48^. The culture supernatant was collected and centrifuged for 1 min at 3000 rpm, after which 100 µL of the supernatant thus obtained was added to 20 µL of the internal standard (1 mg/mL 2-isopropylmalete) and 200 µL acetonitrile and vortexed. After centrifugation for 15 min at 15,000 rpm, 100 µL of each supernatant, thus obtained, was added to a 900 µL MilliQ water and vortexed. Three microliter of the samples was injected into an LC/MS system (ACQUITY UPLC H-Class). The internal (0.125, 0.25, 0.5, and 0.75 mM, 2-isopropylmalete) and external (0.625, 1.25, 2.5, 5, and 10 nM Ado solution) standards were injected together.

### Establishment of transgenic hepatocyte-specific GRAB_ATP_ and GRAB_Ado_ zebrafish

The fabp10-GRAB_ATP_ and fabp10-GRAB_Ado_ plasmids were constructed using PCR with KOD plus Neo (Toyobo) and In-Fusion Snap Assembly Master Mix (Takara) to produce vectors with Tol2 transposon sites. For fabp10-GRAB_ATP_/-GRAB_Ado_, the promoter sequence for hepatocytes was amplified using PCR and used to replace elavl3-GRAB_ATP_/-GRAB_Ado_. Multisite Gateway cloning was performed with the destination vector pDestTol2, the 5′-entry vector containing the fabp10 promoter, the middle entry vector containing pME-mCherry, and the 3′-entry vector containing p3E-polyA. Fabp10:mCherry was used to create fabp10 promoter elements. The zebrafish strains used were as follows: wild-type (AB strain; ZFIN, Eugen, OR, USA), fabp10-GRAB_ATP_, and fabp10-GRAB_Ado_. The plasmid was injected along with the transposon into the one-cell-stage embryo of wild-type zebrafish, as described previously^49^. The plasmid insertion was confirmed by observing heart mCherry in 2 dpf larvae. Injected embryos were raised, and adult zebrafish 2 mpf were identified by amplifying the EGFP gene using PCR with the primers listed in Table 1. They were then outcrossed with wild-type zebrafish to obtain the next generation. Further, to confirm GFP expression in GRAB zebrafish, GRAB_ATP_ and GRAB_Ado_ larvae were embedded in E3 medium with ATP (5 mM) or Ado (6 mM) solutions. Live images were captured as described below.

### Live imaging and GFP detection in GRAB zebrafish

The larvae at 6 or 8 dpf were settled on a glass-bottom dish for live imaging. For imaging, larvae were embedded in 2% low-melting point agarose and time-lapse fluorescence images were acquired with a confocal microscope (FluoView FV3000, Olympus, Tokyo, Japan) using a NA 0.3/10× or 0.5/20× water immersion objective lens. Fluorescence channel and digital image correlation (DIC) images were acquired by sequential line scanning. Z-series were acquired using 208 μm pinhole and 3–4 μm step sizes. Z-series images were stacked using the FluoView FV3000 software (Olympus). To make overlay images of DIC and fluorescence or ratiometric pictures, Z-stacked fluorescence or ratiometric images were overlaid onto a single DIC plane. Finally, the GFP fluorescence sections were examined by setting regions of interest (ROI) at four lesion sites using cellSens (Olympus).

### Histological analysis

Zebrafish were fixed overnight in 4% paraformaldehyde at 4 °C, embedded in paraffin, and processed according to standard procedures. Thereafter, 4-μm sections were stained with HE. All images were obtained using an Axio imager.M2 (Carl Zeiss, Jena, Germany).

### Oil red O staining

Whole larvae were fixed with 4% paraformaldehyde overnight at 4 °C. After fixation, larvae were washed with PBS twice and stained with 0.3% Oil Red O and shaken slowly for 30 min. Stained larvae were washed with PBS-T, followed by two rinses with 60% isopropanol. Then, the zebrafish were transferred to 50% glycerol and placed in 3% methylcellulose, and images were captured using a Leica M205 FA fluorescent stereo microscope (Leica, Wetzlar, Germany).

### mRNA analysis

Total RNA was isolated from pools of 15–20 dissected livers using PureLink™ RNA mini kit (Thermo Fisher Scientific) and a High-Capacity cDNA Reverse Transcription Kit (Thermo Fisher Scientific). qPCR was carried out with KAPA SYBR^®^ Fast qPCR Kit (Kapa Biosystems, Woburn, MA, USA) on a LightCycler 96 System (Roche Diagnostics, Basel, Switzerland) using the following protocol: denaturation at 95 °C for 3 min, followed by 45 cycles of denaturation at 95 °C for 10 s, annealing at 63 °C for 30 s, and extension at 72 °C for 10 s. For analysis of data, the mRNA levels for the target genes were normalized against those of beta-actin, using the comparative threshold method. One-sample *t*-test was performed to compare each treatment with the control for wild-type larvae. The sequences of primers for the selected genes are listed in Table 2.

**Table 2:** Primers used for RT-qPCR of zebrafish gene.

Gene	Forward primer (5′–3′)	Reverse primer (5′–3′)
*β-Actin*	CTGACGGTCAGGTCATCACC	ATGTCCACGTCGCACTTCAT
*tnfa*	GCTTATGAGCCATGCAGTGA	TGCCCAGTCTGTCTCCTTCT
*il1b*	ACTGTTCAGATCCGCTTGCA	TCAGGGCGATGATGACGTTC
*nlrp3*	TCAGCTCTGAGTTCAAACCCC	CACCCATAGGATCAGTTTTGAGTG
*mmp9*	CTCGTTGAGAGCCTGGTGTT	CGCTTCAGATACTCATCCGCT

### Transmission electron microscopy

Larvae, at 8 dpf, were fixed in 1.25% glutaraldehyde and 1% formaldehyde in 0.05 M cacodylate buffer (pH 7.4) for 2 h at 4 °C, followed by postfixing with 2% cacodylate-buffered osmium tetroxide for 2 h at 4 °C. Small tissue blocks were dehydrated with ethanol using a series of ascending concentrations and embedded in epoxy resin. Ultrathin sections were stained with uranyl acetate and lead citrate and observed with an H-7650 transmission electron microscope (Hitachi, Tokyo, Japan).

### Statistical analysis

Data are reported as mean ± standard error of the mean (SEM). Statistical analyses were performed using the GraphPad Prism 9.5.1 software (GraphPad Software). Unpaired two-tailed Student’s *t*-test was used to assess significance when comparing two groups. Statistical significance between three or more groups was determined using two-way analysis of variance (ANOVA) with Tukey’s or Dunnett’s posthoc test. Differences were considered statistically significant at a *P* value < 0.05.

### Supplementary Information

Supplementary Information.

Supplementary Figures.

## Data Availability

The datasets generated during the current study are available from the corresponding author on reasonable request.
