# Supplementary material for: Hepatic extracellular ATP/adenosine dynamics in zebrafish models of alcoholic and metabolic steatotic liver disease
Source: Sci Rep. 2024 Apr 3;14:7813. doi: 10.1038/s41598-024-58043-5 (PMC10987586; doi:10.1038/s41598-024-58043-5)
Supplement: Supplementary file 1 — Supplementary Information. [file 41598_2024_58043_MOESM1_ESM.docx]

**Hepatic extracellular ATP/adenosine dynamics in zebrafish models of alcoholic and metabolic steatotic liver disease**

Tomoko Tokumaru ^1, 2^, Magdeline E. Carrasco Apolinario ^1^, Nobuyuki Shimizu ^3^, Ryohei Umeda ^1, 4^, Koichi Honda ^2^, Kenshiro Shikano ^1^, Hitoshi Teranishi ^1^, Takatoshi Hikida ^5^, Toshikatsu Hanada ^4^, Keisuke Ohta ^6^, Yulong Li ^7^, Kazunari Murakami ^2^ and Reiko Hanada ^1*^

**Supplementary Material and Methods**

To evaluate intracellular and extracellular ATP and adenosine (Ado) levels with or without ethanol (EtOH) or palmitic acid (PA) treatment in HepG2 cells, *in vitro* cell culture experiments were performed following the protocol described in the manuscript. Cells and the supernatant were collected and immediately placed on ice. ATP concentrations were measured using an ATP-Assay-Kit (Dojindo laboratories, Kumamoto, Japan) and Ado concentrations were measured using liquid chromatography with tandem mass spectrometry. Then, clear data that showed discrepancies between intracellular and extracellular ATP and Ado kinetics in the same experiment were obtained (Supplementary Fig. S1 and Fig. S2).

Furthermore, to evaluate the correlation between extracellular ATP or Ado and fluorescence green fluorescent protein (GFP) levels on ATP (100μM) or Ado (1mM) addition to the supernatant in GRAB probe-expressing HepG2 cells, i*n vitro* experiments were performed following the protocols described in the manuscript. Based on this experiment, a clear correlation was confirmed between extracellular ATP or Ado and fluorescence GFP levels (Supplementary Fig. S3).
