# Supplementary material for: Hepatic extracellular ATP/adenosine dynamics in zebrafish models of alcoholic and metabolic steatotic liver disease
Source: Sci Rep. 2024 Apr 3;14:7813. doi: 10.1038/s41598-024-58043-5 (PMC10987586; doi:10.1038/s41598-024-58043-5)
Supplement: Supplementary file 2 — Supplementary Figures. [file 41598_2024_58043_MOESM2_ESM.pptx]

## Slide 1
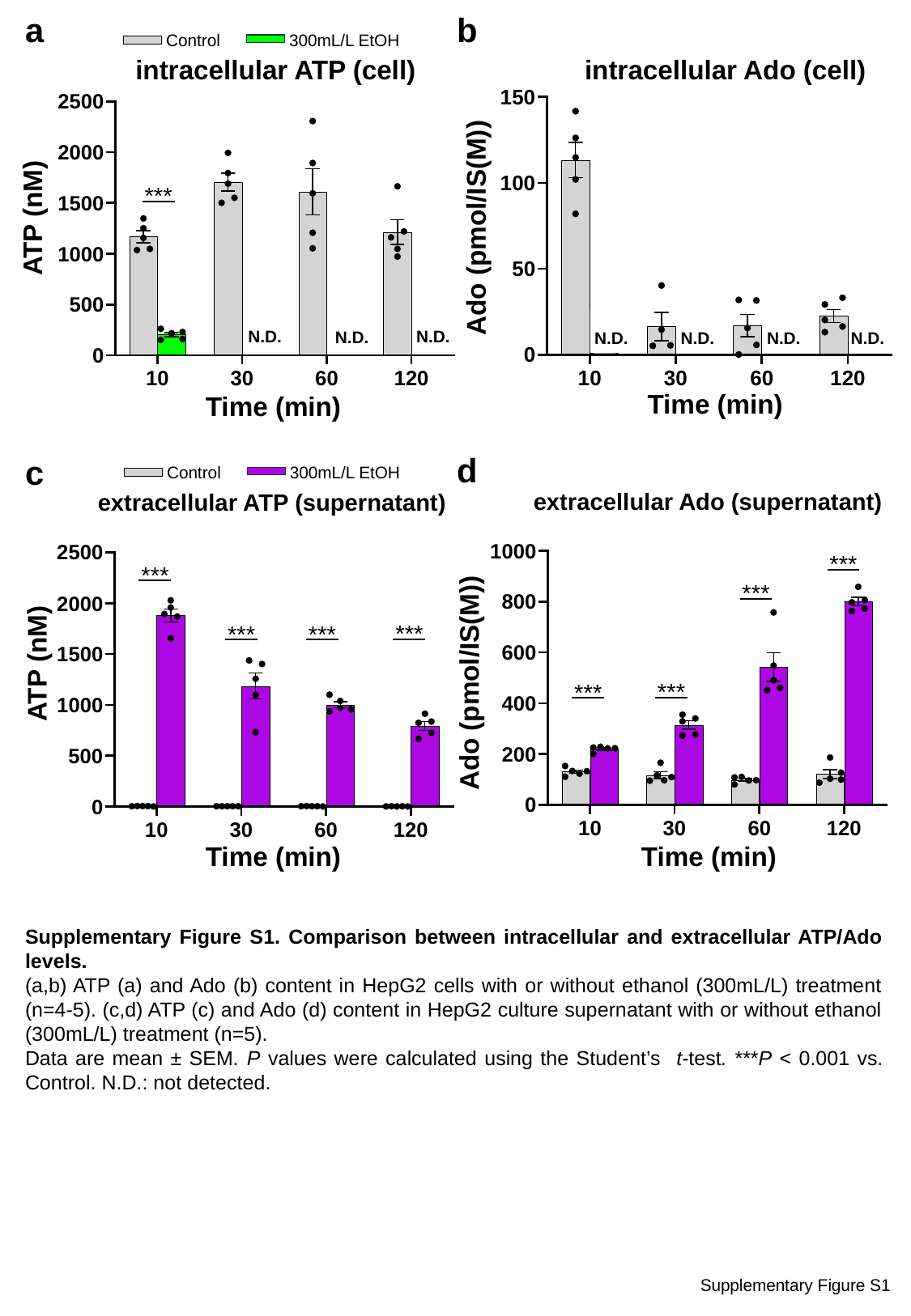

a
b
Control
300mL/L EtOH
intracellular ATP (cell)
intracellular Ado (cell)
***
ATP (nM)
Ado (pmol/IS(M))
N.D.
N.D.
N.D.
N.D.
N.D.
N.D.
N.D.
Time (min)
Time (min)
d
c
Control
300mL/L EtOH
extracellular Ado (supernatant)
extracellular ATP (supernatant)
***
***
***
***
***
***
ATP (nM)
Ado (pmol/IS(M))
***
***
Time (min)
Time (min)
Supplementary Figure S1. Comparison between intracellular and extracellular ATP/Ado levels.
(a,b) ATP (a) and Ado (b) content in HepG2 cells with or without ethanol (300mL/L) treatment (n=4-5). (c,d) ATP (c) and Ado (d) content in HepG2 culture supernatant with or without ethanol (300mL/L) treatment (n=5).
Data are mean ± SEM. P values were calculated using the Student’s t-test. ***P < 0.001 vs. Control. N.D.: not detected.
Supplementary Figure S1

## Slide 2
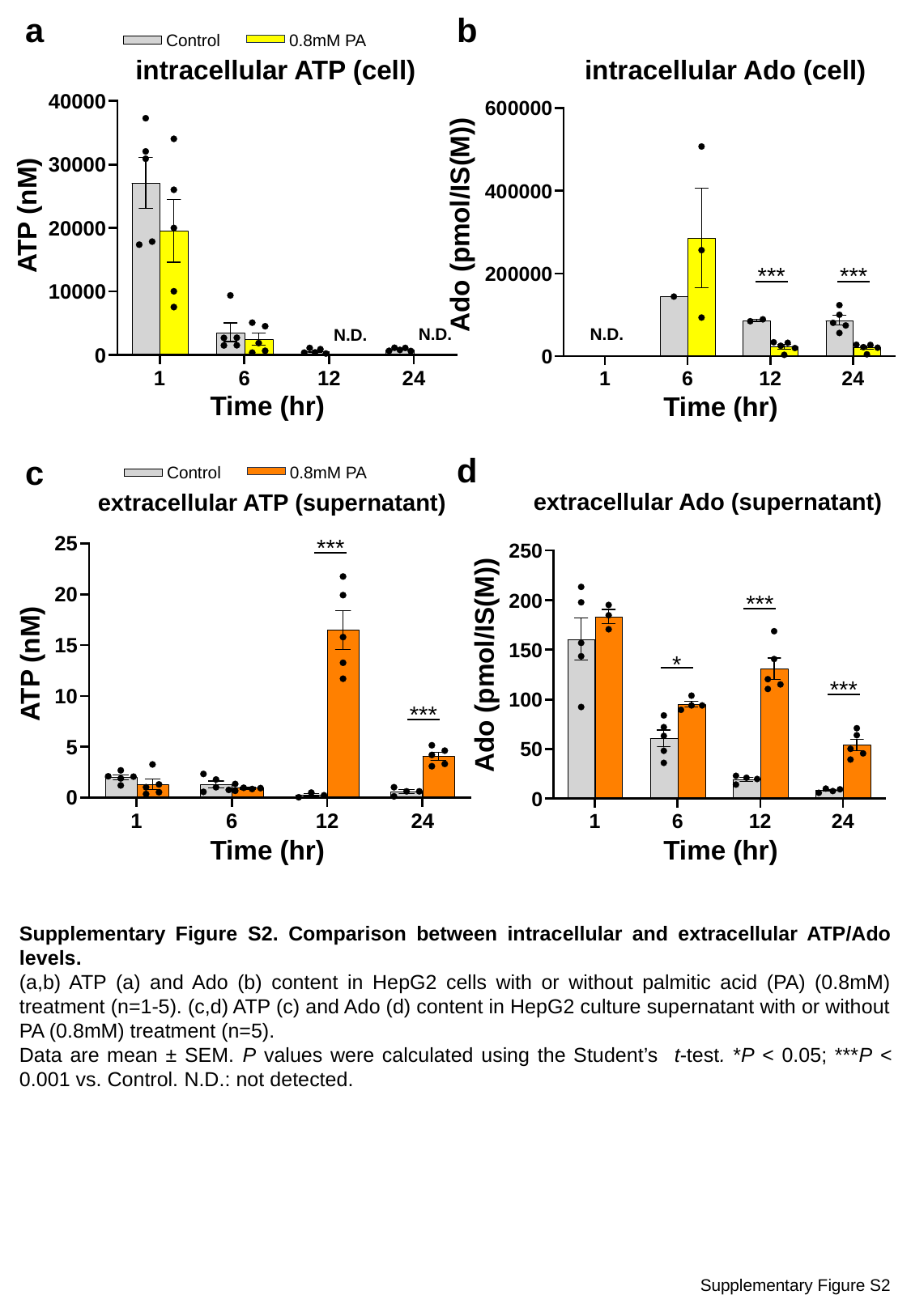

a
b
Control
0.8mM PA
intracellular ATP (cell)
intracellular Ado (cell)
ATP (nM)
Ado (pmol/IS(M))
***
***
N.D.
N.D.
N.D.
Time (hr)
Time (hr)
d
c
Control
0.8mM PA
extracellular Ado (supernatant)
extracellular ATP (supernatant)
***
***
ATP (nM)
Ado (pmol/IS(M))
*
***
***
Time (hr)
Time (hr)
Supplementary Figure S2. Comparison between intracellular and extracellular ATP/Ado levels.
(a,b) ATP (a) and Ado (b) content in HepG2 cells with or without palmitic acid (PA) (0.8mM) treatment (n=1-5). (c,d) ATP (c) and Ado (d) content in HepG2 culture supernatant with or without PA (0.8mM) treatment (n=5).
Data are mean ± SEM. P values were calculated using the Student’s t-test. *P < 0.05; ***P < 0.001 vs. Control. N.D.: not detected.
Supplementary Figure S2

## Slide 3
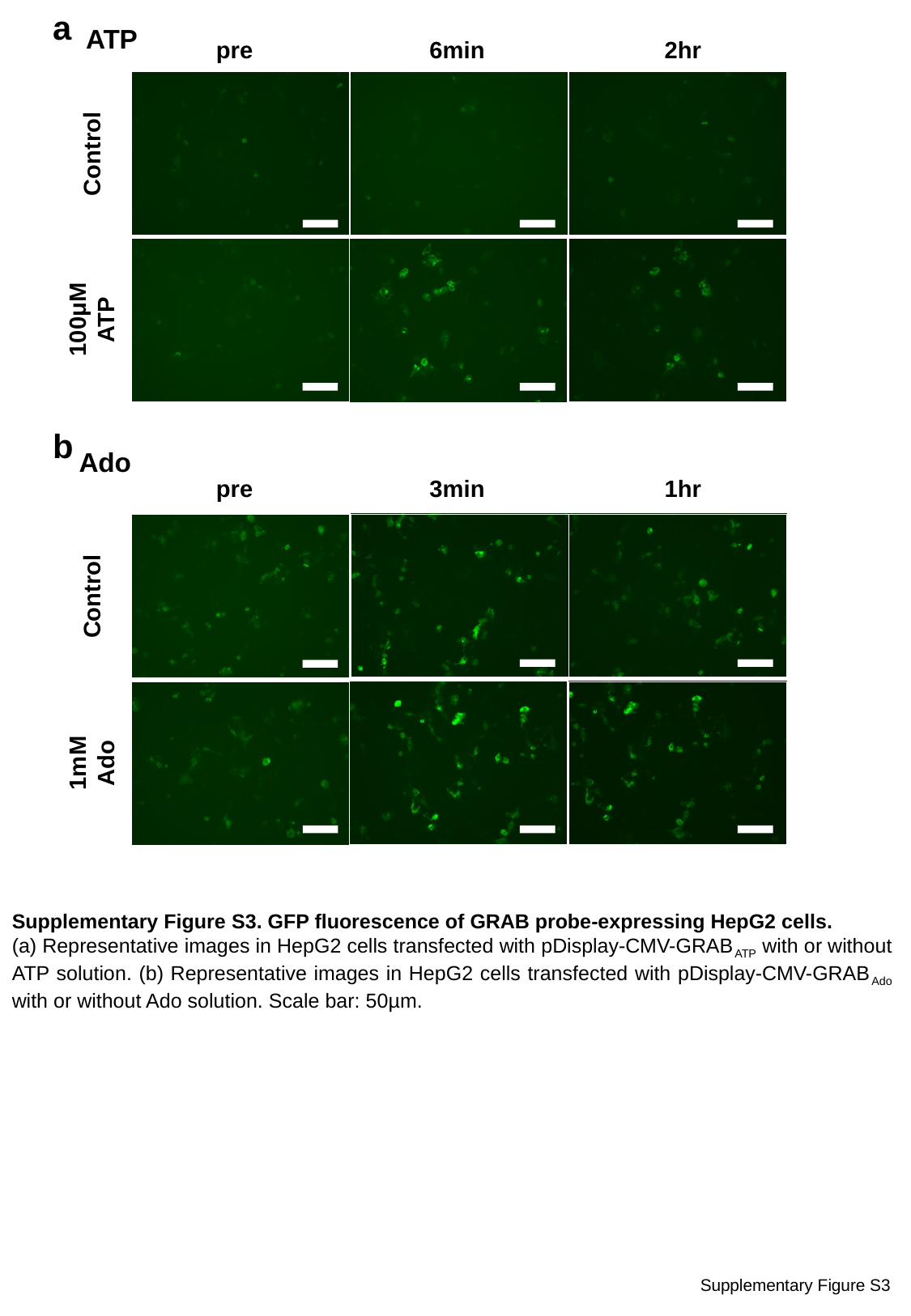

a
ATP
pre
2hr
6min
Control
100μM
ATP
b
Ado
pre
1hr
3min
Control
1mM
Ado
Supplementary Figure S3. GFP fluorescence of GRAB probe-expressing HepG2 cells.
(a) Representative images in HepG2 cells transfected with pDisplay-CMV-GRABATP with or without ATP solution. (b) Representative images in HepG2 cells transfected with pDisplay-CMV-GRABAdo with or without Ado solution. Scale bar: 50µm.
Supplementary Figure S3
